# *Sus scrofa* miR-204 and miR-4331 Negatively Regulate Swine H1N1/2009 Influenza A Virus Replication by Targeting Viral HA and NS, Respectively

**DOI:** 10.3390/ijms18040749

**Published:** 2017-04-03

**Authors:** Shishuo Zhang, Ruifang Wang, Huijuan Su, Biaoxiong Wang, Suolang Sizhu, Zhixin Lei, Meilin Jin, Huanchun Chen, Jiyue Cao, Hongbo Zhou

**Affiliations:** 1 State Key Laboratory of Agricultural Microbiology, College of Veterinary Medicine, Huazhong Agricultural University, Wuhan 430070, China; zhangshishuo006@126.com (S.Z.); wangruifang29@163.com (R.W.); shj741984661@126.com (H.S.); wangbiaoxiong@126.com (B.W.); lei460411658@163.com (Z.L.); jml8328@126.com (M.J.); chenhch@mail.hzau.edu.cn (H.C.); caojiyue@mail.hzau.edu.cn (J.C.); 2 Department of Animal Science, Tibet Agricultural and Animal Husbandry College, Linzhi 860000, China; xzslsz@163.com; 3 The Cooperative Innovation Center for Sustainable Pig Production, Wuhan 430070, China

**Keywords:** virus replication, viral genomic RNA, *Sus scrofa* miR-204, *Sus scrofa* miR-4331, RegRNA 2.0, miRNA-virus interaction

## Abstract

The prevalence of swine pandemic H1N1/2009 influenza A virus (SIV-H1N1/2009) in pigs has the potential to generate novel reassortant viruses, posing a great threat to human health. Cellular microRNAs (miRNAs) have been proven as promising small molecules for regulating influenza A virus replication by directly targeting viral genomic RNA. In this study, we predicted potential *Sus scrofa* (ssc-, swine) miRNAs targeting the genomic RNA of SIV-H1N1/2009 by RegRNA 2.0, and identified ssc-miR-204 and ssc-miR-4331 to target viral HA and NS respectively through dual-luciferase reporter assays. The messenger RNA (mRNA) levels of viral HA and NS were significantly suppressed when newborn pig trachea (NPTr) cells respectively overexpressed ssc-miR-204 and ssc-miR-4331 and were infected with SIV-H1N1/2009, whereas the suppression effect could be restored when respectively decreasing endogenous ssc-miR-204 and ssc-miR-4331 with inhibitors. Because of the importance of viral HA and NS in the life cycle of influenza A virus, ssc-miR-204 and ssc-miR-4331 exhibited an inhibition effect on SIV-H1N1/2009 replication. The antiviral effect was sequence-specific of SIV-H1N1/2009, for the target sites in HA and NS of H5N1 or H9N2 influenza A virus were not conserved. Furthermore, SIV-H1N1/2009 infection reversely downregulated the expression of ssc-miR-204 and ssc-miR-4331, which might facilitate the virus replication in the host. In summary, this work will provide us some important clues for controlling the prevalence of SIV-H1N1/2009 in pig populations.

## 1. Introduction

Influenza viruses, which belong to the orthomyxovirus family, are enveloped, single-stranded and negative-sense RNA viruses [1]. The segmented genome of influenza A virus is composed of eight different viral RNA segments, each encoding one or two different viral proteins [2]. In addition to ten common viral proteins (PB1, PB2, PA, NP, HA, NA, M1, M2, NS1 and NS2), there are several newly identified proteins generated by various co-transcriptional or co-translational strategies, including PB1-F2 [3], PB1-N40 [4], PA-X [5], N-truncated PAs [6], M42 [7], and NS3 [8]. All viral proteins play important roles in the life cycle of influenza viruses. Based on the two surface glycoproteins HA and NA, influenza A viruses are divided into different subtypes. To date, 16 types of HA and 9 types of NA have been identified in viruses from wild birds, while two influenza-like viruses H17N10 and H18N11 were recently identified from bats [9]. More than 100 possible HA–NA combinations have been found in nature [10], for the influenza A virus genome can undergo genetic reassortment, enabling the virus to infect a wide range of hosts.

Influenza A viruses are among the most important human pathogens that cause yearly epidemics and occasional pandemics. Each year, the global burden of influenza epidemics is 3–5 million cases of severe illness. Each influenza pandemic results in more serious social and economic impact. A novel H1N1 influenza A virus, which emerged in Mexico and the United States in March and early April 2009, caused the first influenza pandemic of the 21st century [11]. The virus was found to be genetically and antigenically unrelated to human seasonal influenza viruses [12], and phylogenetic analyses showed that it probably resulted from the reassortment of North American H3N2 and H1N2 swine viruses with Eurasian avian-like swine viruses [13]. By the end of April, the World Health Organization (WHO) had to increase the pandemic alert from phase 3 to phase 4, and shortly after to phase 5 because of the international spread and clusters of human-to-human transmission [12]. According to an announcement of the official end of the pandemic, the virus had spread to more than 200 countries and had caused more than 18,000 human deaths worldwide by August 2010 [14]. Soon, the pandemic H1N1/2009 virus was also isolated from a swine herd firstly in Canada [15], and subsequently in China [16,17], Thailand [18], South Korea [19], the United Kingdom [20] and others. It was worth noting that pigs were considered as the hypothetical “mixing vessel” in which human and avian viruses could reassort, because both human- and avian-type influenza A virus receptors could be expressed on swine epithelial cells in trachea [21]. As a result, novel viruses were generated by the pandemic H1N1/2009 virus with other influenza viruses circulating in pig populations [22,23,24], which might produce the potential threat to public health. Therefore, it is very important to surveil and control the swine pandemic H1N1/2009 influenza A virus (SIV-H1N1/2009).

MicroRNAs (miRNAs), a class of ~22 nucleotide long non-coding RNAs, are considered as crucial regulators for modulating genes expression by binding to the messenger RNA (mRNA), resulting in target cleavage or translational repression depending on the extent of sequence complementarity [25]. Reported studies demonstrated that miRNAs played an important role in a broad spectrum of host biological processes, including differentiation and proliferation [26], development [27], apoptosis [28], and viral infections [29]. Recently, the roles of miRNAs in intricate host–virus interaction networks have gained more and more attention in the case of influenza A virus. Several studies suggested that miRNAs acted in the host antiviral response by altering the expression of host genes required for virus replication [30,31], or by regulating the immune signaling pathways [32,33], or via directly targeting influenza virus genomic RNA [29,33,34,35,36]. Among these, the most effective mechanism of miRNAs mediating antiviral defense might be targeting the viral genomic RNA.

However, there were few studies underlying the interactions of *Sus scrofa* (ssc-, swine) miRNAs and swine influenza viruses. In 2010, our lab isolated a pandemic H1N1/2009 influenza A virus in swine herds [17], and the next work demonstrated that this swine influenza virus possessed enhanced pathogenicity in mice [37]. In order to explore swine miRNAs that could regulate the SIV-H1N1/2009 replication, we predicted potential miRNAs targeting the viral genomic RNA by bioinformatics method, and identified ssc-miR-204 and ssc-miR-4331 as negative regulators of SIV-H1N1/2009 replication by targeting viral HA and NS respectively.

## 2. Results

### 2.1. Prediction of Potential Swine miRNAs Targeting SIV-H1N1/2009 Genomic RNA

Cellular miRNAs were shown to play an important role in the life cycle of influenza A virus by directly targeting the viral genomic RNA. In order to investigate which swine miRNAs were involved in mediating the host and SIV-H1N1/2009 direct interaction, computational screening for potential miRNAs that targeted the viral genomic RNA was performed. RegRNA 2.0, an online server integrating miRanda and miRBase, was used for prediction, and the cutoff value was set as “score ≥ 150.00 and free-energy ≤ −20.00”. As shown in Table 1, there were 2, 6, 2, 5, 1, 4 and 2 miRNAs predicted to target viral PB1, PB2, PA, HA, NA, NP and NS of SIV-H1N1/2009, respectively. No binding sites for any miRNAs in the viral M gene were predicted by RegRNA 2.0. Interestingly, some miRNAs were found to target more than one gene of SIV-H1N1/2009, for example, the binding sites for ssc-miR-361-3p located in both viral PB2 and HA, and the binding sites for ssc-miR-136 located in both viral NA and NP.

**Figure 1:**
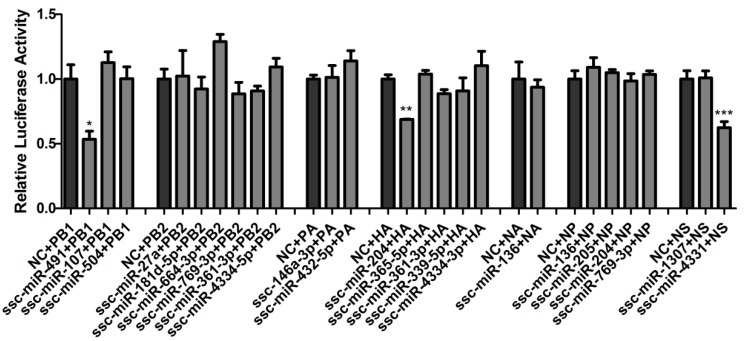
Overall validation of potential miRNAs targeting SIV-H1N1/2009 genomic RNA by dual-luciferase reporter arrays. Seven genes of SIV-H1N1/2009 were cloned into the luciferase reporter vector pmirGLO and then cotransfected with their corresponding predicted miRNA in newborn pig trachea (NPTr) cells. The cells were harvested for assaying luciferase activity at 24 h post-transfection. The pair of ssc-miR-491 and PB1 was considered as the positive control. Data were shown as relative firefly luciferase activities normalized to the value of renilla luciferase. Significant levels were analyzed by Student’s *t*-test, * *p* ≤ 0.05, ** *p* ≤ 0.01, *** *p* ≤ 0.001. NC: negative controls.

Next, the preliminary validation of these potential miRNAs targeting SIV-H1N1/2009 genomic RNA was carried out by dual-luciferase reporter arrays. The above seven genes of SIV-H1N1/2009 were cloned into the luciferase reporter vector pmirGLO and then cotransfected with their corresponding miRNAs mimics in newborn pig trachea (NPTr) cells. At 24 h post-transfection, the cells were harvested for assaying luciferase activity. However, the relative luciferase activity was only decreased when the cells were cotransfected with ssc-miR-204 and pmirGLO-HA or ssc-miR-4331 and pmirGLO-NS, but remained unchanged when the cells were cotransfected with other pairs of miRNA-genes (Figure 1), which indicated that ssc-miR-204 and ssc-miR-4331 might target viral HA and NS of SIV-H1N1/2009, respectively. As miR-491 had been verified to target viral PB1 of A/WSN/33 [34], and the binding sites were conserved with that of the ssc-miR-491 in viral PB1 of SIV-H1N1/2009, the pair of ssc-miR-491 and PB1 was considered as the positive control.

### 2.2. Validation of ssc-miR-204 and ssc-miR-4331 Targeting Viral HA and NS of SIV-H1N1/2009, Respectively

**Figure 2:**
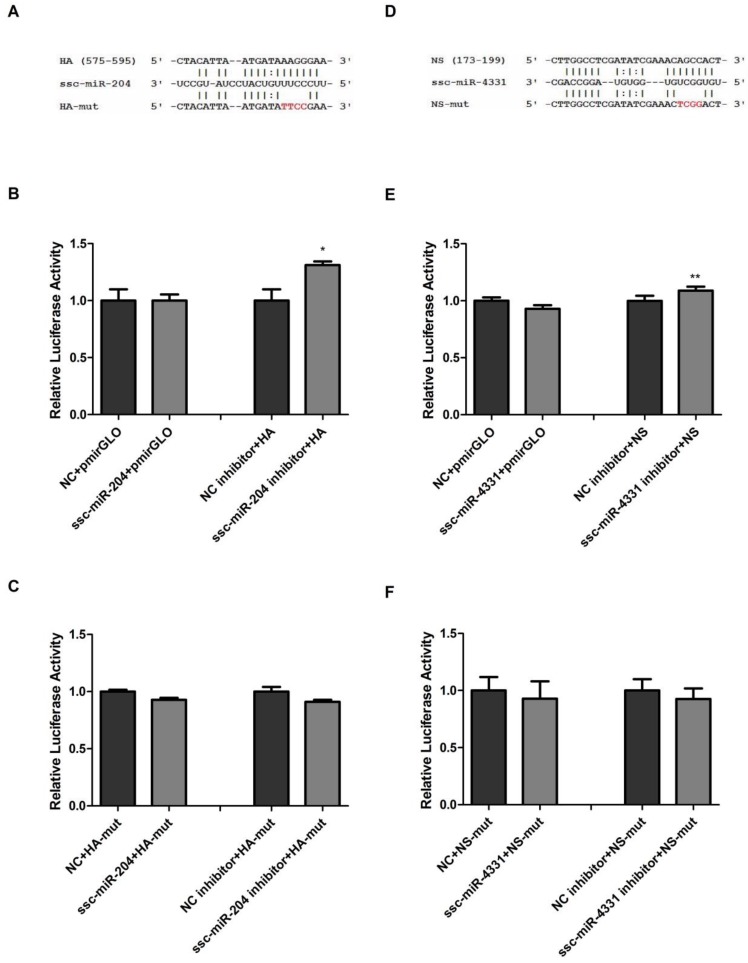
Ssc-miR-204 and ssc-miR-4331 targeted viral HA and NS of SIV-H1N1/2009, respectively. Sequence alignments of ssc-miR-204 and its target sites in viral HA (**A**); and that of ssc-miR-4331 and its target sites in viral NS (**D**). Four mutated nucleotides of the target sites were indicated in red. NPTr cells were firstly cotransfected with the luciferase reporter vector pmirGLO (**B**,**E**) or wild-type reporter pmirGLO-HA (**B**,**E**) or the mutated reporter pmirGLO-HA-mut (**C**,**F**) with the indicated RNA oligonucleotides, then the cell lysates were harvested for dual-luciferase reporter assays 24 h post-transfection. Data were shown as relative firefly luciferase activities normalized to the value of renilla luciferase. Significant levels were analyzed by Student’s *t*-test, * *p* ≤ 0.05, ** *p* ≤ 0.01.

In order to exclude the possibility of ssc-miR-204 and ssc-miR-4331 targeting the luciferase, we cotransfected ssc-miR-204 or ssc-miR-4331 with the luciferase reporter vector pmirGLO into NPTr cells and found that the relative firefly luciferase activities were not changed significantly (Figure 2B,E). Then, we cotransfected ssc-miR-204 inhibitors with the reporter construct pmirGLO-HA into NPTr cells and found the relative firefly luciferase activity was increased significantly compared with that of control cells (Figure 2B). A similar effect was observed after the cells being cotransfected with ssc-miR-4331 and pmirGLO-NS (Figure 2E). These results further indicated the target relationship of ssc-miR-204 and HA, and ssc-miR-4331 and NS. Furthermore, the binding sites for ssc-miR-204 and ssc-miR-4331 were predicted to be located at nt 575–595 in HA and nt 173–199 in NS respectively (Figure 2A,D). Four base pair mutations (in red) in the seed regions of HA or NS were introduced into the corresponding wild-type luciferase reporters, resulting in pmirGLO-HA-mut and pmirGLO-NS-mut. As expected, the decrease or increase trend of luciferase activity was completely abrogated when NPTr cells were treated with pmirGLO-HA-mut together with ssc-miR-204 mimics or inhibitors (Figure 2C), so was the pair of pmirGLO-NS-mut and ssc-miR-4331 (Figure 2F). Taken together, these results validated that ssc-miR-204 and ssc-miR-4331 directly interacted with the corresponding HA and NS of SIV-H1N1/2009, and the target sites were located in HA1 and NS1, respectively.

### 2.3. Ssc-miR-204 and ssc-miR-4331 Repressed the Expression of Viral HA1 and NS1 Respectively, and Inhibited the Replication of SIV-H1N1/2009

**Figure 3:**
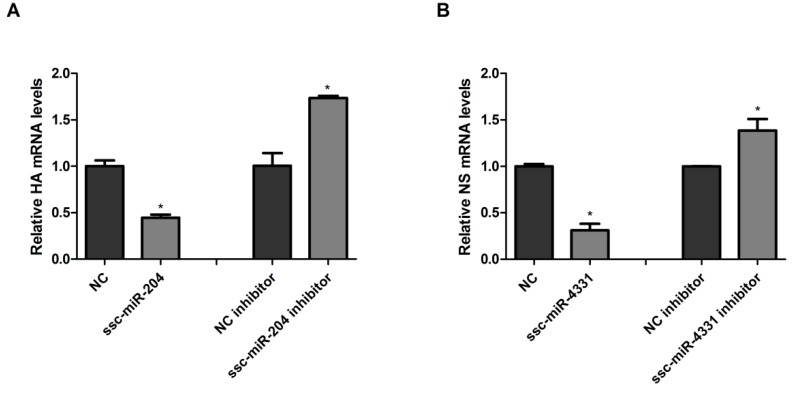
Ssc-miR-204 and ssc-miR-4331 suppressed the expression of SIV-H1N1/2009 HA and NS, respectively. (**A**) NPTr cells were transfected with ssc-miR-204 mimics or inhibitors for 24 h and infected with SIV-H1N1/2009 for 36 h, then the mRNA levels of viral HA were assessed by quantitative real-time PCR (qRT-PCR) method; (**B**) NPTr cells were transfected with ssc-miR-4331 mimics or inhibitors for 24 h and infected with SIV-H1N1/2009 for 36 h, then the mRNA levels of viral NS were assessed by qRT-PCR method. All data were determined using the 2^−ΔΔ*C*t^ method and normalized to glyceraldehyde-3-phosphate dehydrogenase (GAPDH). Significant levels were analyzed by Student’s *t*-test, * *p* ≤ 0.05.

According to the reported studies, miRNAs regulated genes expression at posttranscriptional level by directly targeting the genes. ssc-miR-204 and ssc-miR-4331 were validated to target viral HA1 and NS1 of SIV-H1N1/2009 respectively, then the effect of both miRNAs on the expression of HA1 and NS1 were investigated. NPTr cells overexpressed ssc-miR-204 or ssc-miR-4331 by being transfected with the corresponding miRNA mimics and were infected with SIV-H1N1/2009, then total RNA was extracted and reverse transcribed to cDNA which was subjected to quantitative real-time PCR (qRT-PCR). As shown in Figure 3A,B, the ectopic expression of ssc-miR-204 or ssc-miR-4331 markedly suppressed the expression of viral HA1 or NS1, respectively, at mRNA levels. A significant increase of viral HA1 or NS1 mRNA expression was observed when endogenous ssc-miR-204 or ssc-miR-4331 was decreased by treating with the corresponding miRNA inhibitors.

**Figure 4:**
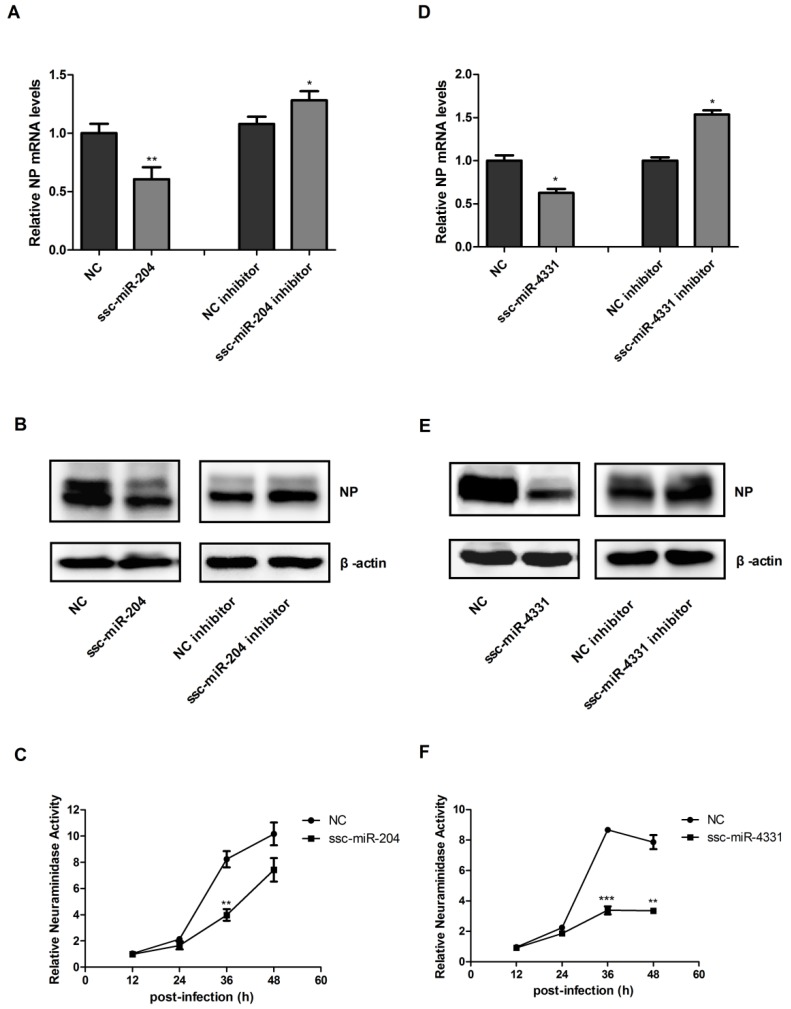
Ssc-miR-204 and ssc-miR-4331 inhibited SIV-H1N1/2009 replication. NPTr cells were transfected with ssc-miR-204/ssc-miR-4331 mimics or inhibitors for 24 h and then infected with SIV-H1N1/2009. The cells were collected at 36 h post-infection to detect the mRNA levels (**A**,**D**) and protein levels (**B**,**E**) of viral NP using qRT-PCR and western blotting respectively; (**C**,**F**) The neuraminidase activities of the supernatants were measured using a 2′-(4-methylumbelliferyl)-α-d-*N*-acetylneuraminic acid (MUNANA) assay at 12, 24, 36 and 48 h post-infection. Values were normalized to the neuraminidase activity measured in supernatant of control cells at 12 h post-infection. Significant levels were analyzed by Student’s *t*-test, * *p* ≤ 0.05, ** *p* ≤ 0.01, *** *p* ≤ 0.001.

While HA and NS are important parts of viral genome of SIV-H1N1/2009, ssc-miR-204 and ssc-miR-4331 may negatively regulate the virus replication. In order to verify this speculation, NPTr cells were transfected with ssc-miR-204 mimics or inhibitors and infected with SIV-H1N1/2009, then the mRNA and protein levels of NP (a conserved viral protein of influenza A virus) were detected using qRT-PCR and western blotting, respectively. The virus titers of swine H1N1/2009 influenza A virus were evaluated by measuring the neuraminidase activity of the cells supernatant using a 2′-(4-methylumbelliferyl)-α-d-*N*-acetylneuraminic acid (MUNANA) assay. As shown in Figure 4A,B, the expression of NP was repressed significantly at both mRNA and protein levels in ssc-miR-204 overexpressed cells compared with the control cells, whereas the NP levels were upregulated markedly in cells transfected ssc-miR-204 inhibitors. The neuraminidase activities of cells supernatants were decreased by ssc-miR-204 from 24 h post-infection, especially, a huge drop was observed at 36 h. Similar results were obtained when cells were treated with ssc-miR-4331 mimics or inhibitors (Figure 4D–F). In summary, the above results suggested that ssc-miR-204 and ssc-miR-4331 suppressed viral HA and NS expression by directly interacting with them respectively, resulting in inhibiting the replication of SIV-H1N1/2009.

### 2.4. The Effect of ssc-miR-204 and ssc-miR-4331 on the Replication of H5N1 or H9N2 Influenza A Virus

**Figure 5:**
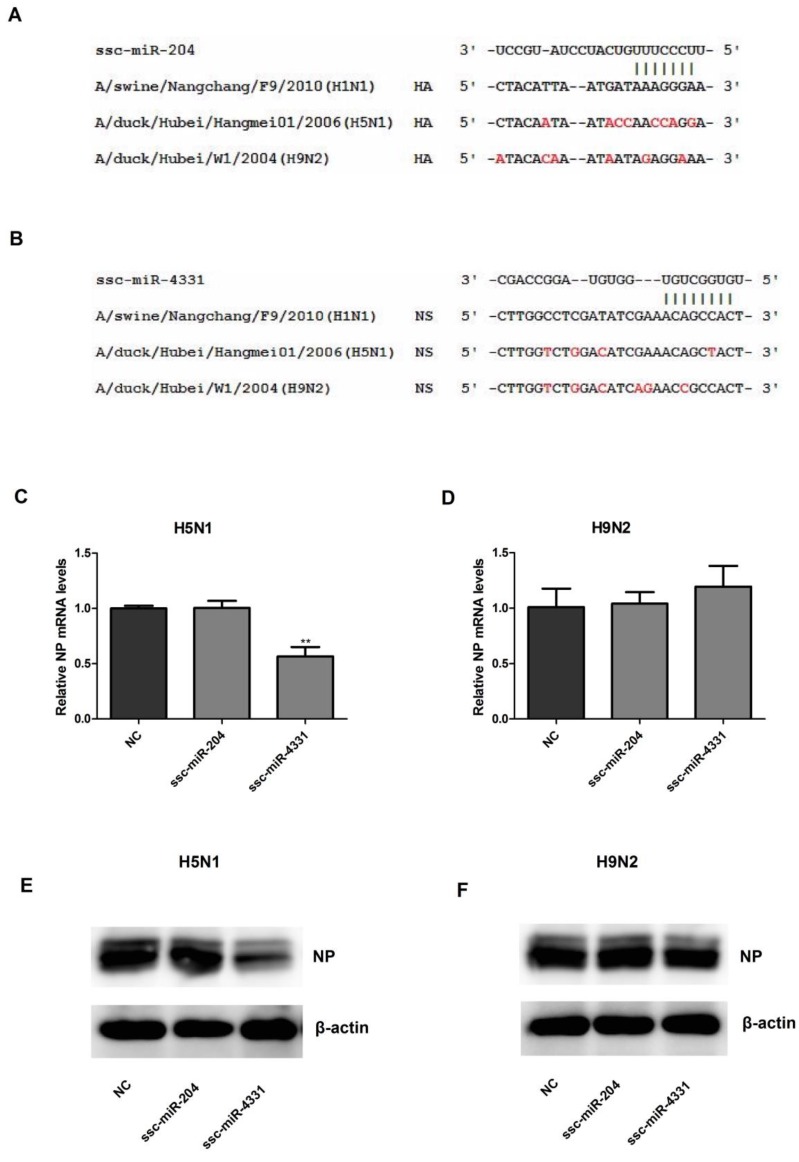
The effect of ssc-miR-204 and ssc-miR-4331 on the replication of H5N1 or H9N2 influenza A virus. (**A**) Sequence alignments of ssc-miR-204 and its target sites in viral HA of three subtypes of influenza A virus (H1N1, H5N1 and H9N2); (**B**) Sequence alignments of ssc-miR-4331 and its target sites in viral NS of three subtypes of influenza A virus (H1N1, H5N1 and H9N2). NPTr cells were transfected with ssc-miR-204 or ssc-miR-4331 for 24 h, then infected with H5N1 or H9N2 influenza A virus for 24 h. The mRNA levels of H5N1 NP (**C**) or H9N2 NP (**D**) were assessed by qRT-PCR method. All data were determined using 2^−ΔΔ*C*t^ and normalized to GAPDH. Significant levels were analyzed by Student’s *t*-test, ** *p* ≤ 0.01. The protein levels of H5N1 NP (**E**) or H9N2 NP (**F**) were assessed by western blotting. NP expressions were normalized to β-actin.

To investigate whether the antiviral mechanism of ssc-miR-204 and ssc-miR-4331 was broad spectrum among various subtypes of influenza A virus, H5N1 and H9N2 were chosen as the representatives to assess the antiviral effect of both the miRNAs. As shown in Figure 5A,B, the target sites in viral HA and NS for the corresponding seed regions of ssc-miR-204 and ssc-miR-4331 were aligned among H1N1, H5N1 and H9N2 influenza A viruses. However, the alignment results showed that the sequences of the target sites in viral HA and NS were not conserved across the three different representative subtypes, which indicated that ssc-miR-204 and ssc-miR-4331 might not inhibit the replication of H5N1 or H9N2 influenza A virus by targeting HA and NS respectively. In order to address this speculation, NPTr cells were transfected with ssc-miR-204 or ssc-miR-4331 and infected with H5N1 or H9N2 influenza A virus, then the mRNA and protein levels of viral NP were detected using qRT-PCR and western blotting method, respectively. As expected, no suppression effect of ssc-miR-204 and ssc-miR-4331 on H9N2 NP expression at mRNA levels (Figure 5D) nor at protein levels (Figure 5F), was observed, which proved that both miRNAs did not have an effect on the replication of H9N2 influenza A virus. Similarly, ssc-miR-204 was found to have no influence on the mRNA and protein levels of H5N1 NP (Figure 5C,E), which indicated that it did not inhibit the replication of H5N1 influenza A virus either. Meanwhile, ssc-miR-4331 was detected to significantly decrease the mRNA levels (Figure 5C) and protein levels (Figure 5E) of H5N1 NP unexpectedly. In order to explore whether ssc-miR-4331 inhibited the H5N1 influenza A virus replication by targeting other viral genes, computational screening for potential miRNAs targeting the whole genome of H5N1 influenza A virus was performed. However, there were no target sites of ssc-miR-4331 in the eight viral genes of H5N1 influenza A virus (Table 2).

### 2.5. SIV-H1N1/2009 Infection Downregulated the Expression of ssc-miR-204 and ssc-miR-4331

**Figure 6:**
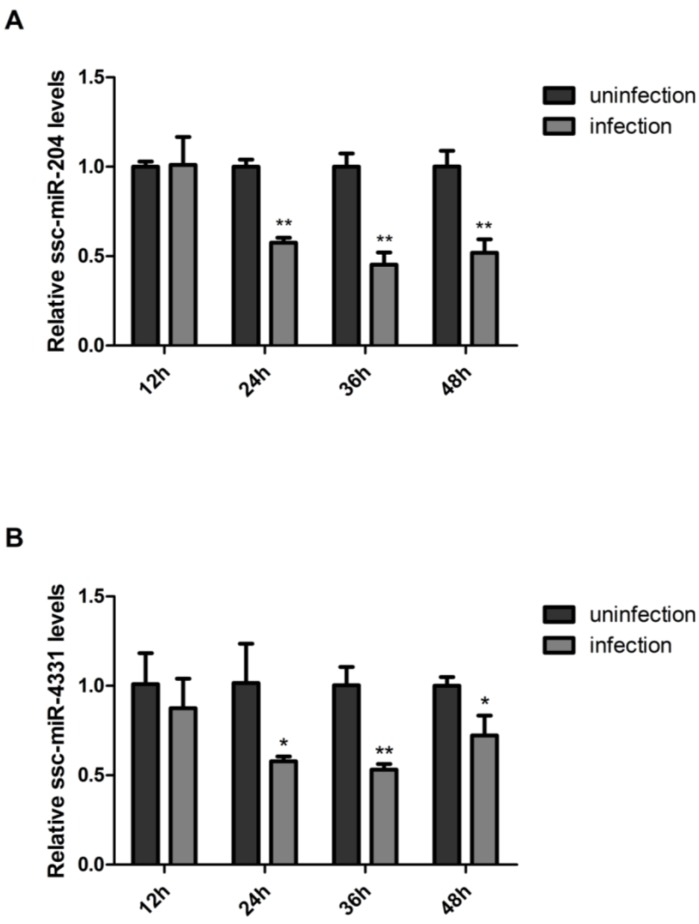
SIV-H1N1/2009 infection downregulated the expression of ssc-miR-204 and ssc-miR-4331. NPTr cells were infected with SIV-H1N1/2009 and harvested at 12, 24, 36 and 48 h post-infection, respectively. Total cellular RNA was extracted and subjected to RT reaction. The expression levels of ssc-miR-204 (**A**) and ssc-miR-204 (**B**) were determined by qRT-PCR methods. All data were standardized to 1 in uninfected cells for each time point. Significant levels were analyzed by Student’s *t*-test, * *p* ≤ 0.05, ** *p* ≤ 0.01.

Since ssc-miR-204 and ssc-miR-4331 negatively regulated the replication of SIV-H1N1/2009, we wondered whether the virus had an impact on the miRNAs expression. Therefore, the abundant change of both miRNAs in response to the virus infection was investigated. Firstly, NPTr cells were infected or uninfected with SIV-H1N1/2009, and collected at indicated time points post-infection. Then, the mRNA levels of ssc-miR-204 and ssc-miR-4331 were subsequently quantified by qRT-PCR method. As shown in Figure 6A, the ssc-miR-204 level did not present significant change at 12 h post-infection, but from 24 h, a marked reduction was observed in infected cells compared with uninfected cells, with the same occurring for ssc-miR-4331 levels in response to SIV-H1N1/2009 infection (Figure 6B). In conclusion, this result suggested that SIV-H1N1/2009 might weaken the antiviral effect of ssc-miR-204 and ssc-miR-4331 by decreasing their expression levels to facilitate its survival in the host during the infection process.

## 3. Discussion

The pandemic H1N1/2009 influenza A virus caused the first influenza pandemic of the 21st century, resulting in the infection of millions of people and producing great economic losses and social panic worldwide. The virus was firstly announced to be found in two children in southern California by the US Centers for Disease Control and Prevention in April 2009 [38]. Soon thereafter, it was also isolated from a swine herd in Alberta, Canada in May 2009 [15]. More seriously, the pandemic H1N1/2009 influenza A virus was reported to reassort with other influenza virus strains into novel viruses in swine populations afterwards. As Vijaykrishna’s group reported, a novel reasortant, A/swine/HongKong/201/2010 (H1N1), appeared with a pandemic H1N1/2009-like NA gene [22]. In Moreno’s study, the phylogenetic analysis of a novel virus isolation, A/Sw/It/116114/2010 (H1N2), showed that all of the genes belonged to the pandemic H1N1/2009 cluster with the exception of NA [23]. In China, one group of novel reassortants possessing pandemic H1N1/2009 influenza A virus internal genes was also isolated. Animal experiments showed that the virus transmitted effectively from pigs to pigs and from pigs to ferrets, and it could also replicate in ex vivo human lung tissue [24]. In a word, the continuing prevalence of the pandemic H1N1/2009 influenza A virus in pigs could lead to generate novel swine reassortant viruses with the potential threat to public health. However, the current drugs approved for treatment or prevention of influenza only acted on M2 (amantadine and rimantadine) and NA (oseltamivir, zanamivir and peramivir). Widespread resistance to these drugs in current circulating viruses had emerged. Therefore, it was necessary to find new potential targets for antiviral drugs to control swine pandemic H1N1/2009 influenza A virus.

In recent years, miRNAs have been recognized as promising small molecules for developing new antiviral drugs. Several miRNAs were reported to directly target viral genomic RNA of influenza A virus. In 2010, Song’s group reported that miR-323, miR-491 and miR-654 could target the viral PB1 gene [34]. Later in 2012, miR-3145 was also reported to interact with viral PB1 gene [36]. Then in recent 2015, another miRNA miR-485 was proven to bind the viral PB1 gene as the same [33]. In addition, miR-let-7c was demonstrated to target the M1 gene of influenza A virus, which was different from the above miRNAs targeting the PB1 gene [35]. However, no miRNAs were verified to interact with viral HA or NS gene to our knowledge. Only miR-145 was predicted to have putative target sites in the HA gene [39], and an unpublished data mentioned that miR-136 also had potential binding sites in the HA gene [29]. In this work, overall screening for potential miRNA-targeting genomic RNA of SIV-H1N1/2009 was performed using RegRNA 2.0. For the first time, ssc-miR-204 and ssc-miR-4331 were verified to target viral HA and NS respectively by dual-luciferase reporter assays. The HA protein is the surface glycoprotein mediating the binding of the virus to host cells and the subsequent fusion of the viral and endosomal membranes for vRNP release into the cytoplasm [40]. The NS1 protein is an interferon antagonist that blocks the activation of transcription factors and IFN-β-stimulated gene products [41]. Because of their importance in virus pathogenicity, ssc-miR-204 and ssc-miR-4331 were demonstrated to negatively regulate the replication of SIV-H1N1/2009 by targeting viral HA and NS respectively and repressing their expression levels.

However, this antiviral effects of ssc-miR-204 and ssc-miR-4331 were sequence-specific for SIV-H1N1/2009. In our work, alignment of the binding sites in the viral HA and NS genes for the corresponding seed region of ssc-miR-204 and ssc-miR-4331 was performed among different subtypes (H1N1, H5N1 and H9N2) of influenza A viruses, and results revealed that the sequences of target sites were not conserved, which indicated that ssc-miR-204 or ssc-miR-4331 did not target the corresponding H5N1 and H9N2 viral HA or NS genes to repress their expression. As expected, neither of the miRNAs could inhibit the replication of H9N2 influenza A virus effectively, with the same occurring for ssc-miR-204 on H5N1 replication. Referring to the reason why ssc-miR-4331 exhibited the antiviral effect on the replication of H5N1 influenza A virus, it might be due to ssc-miR-4331 targeting and regulating some host genes by as yet unknown mechanisms, for ssc-miR-4331 was predicted not to target other viral genes of H5N1 influenza A virus either. However, three putative interactions between swine miRNAs and swine influenza A virus were found to be maintained almost throughout all the swine virus evolution in He’s study [39]. They collected viral sequences isolated in 38 different years and predicted the miRNA-target pairs by miRanda software. Ssc-miR-124a was found to target the NP genes isolated in all 38 different times, while ssc-miR-145 targeted the NP genes isolated at 35 different times and ssc-miR-136 targeted the NA and NP genes isolated in 31 of the 38 times. Interestingly, our genomic RNA screen also predicted the interaction pairs of ssc-miR-136 and NA, and ssc-miR-136 and NP. However, they were proven as false-positive predictions by the dual-luciferase reporter assays. In addition, the interaction of ssc-miR-124a and NP, and ssc-miR-145 and NP were not predicted in our work, which might be due to the different prediction tools and criteria, or the sequence-specificity of SIV-H1N1/2009.

Furthermore, the influenza A virus infection can reversely alter the miRNAs expression levels in the host. In our work, ssc-miR-204 and ssc-miR-4331 were downregulated by SIV-H1N1/2009 infection. However, chicken miR-204 was highly expressed in infected chicken lungs by low pathogenic H5N3 influenza A virus [42]. Human miR-204 was also upregulated in whole blood of H1N1 patients [43]. This discrepancy might be due to that miR-204 were encoded by different hosts infected by different strains of influenza A viruses, and detected at different time points. For ssc-miR-4331, it was reported to be detected in porcine intestinal [44], liver [45] and milk [46], and it could be upregulated by transmissible gastroenteritis virus (TGEV) infection and inhibit transcription of TGEV gene 7 via directly targeting cell division cycle-associated protein 7 [47]. However, few studies revealed ssc-miR-4331 to be involved in influenza A viruses infection to our knowledge. Therefore, our work was the first to illustrate the expression profile and function of ssc-miR-4331 in response to influenza A virus infection.

There are some limitations that need to be addressed and resolved in our future work. Firstly, almost no tool but RegRNA2.0 could consider the species specificity in the miRNA prediction to our knowledge, so we only used RegRNA2.0 to predict the potential swine miRNAs targeting the genomic RNA of SIV-H1N1/2009, which might lead to the false positive or false negative in our prediction work. Secondly, the protein levels of viral HA and NS were not assessed after ssc-miR-204 and ssc-miR-4331 treatment respectively, for we did not obtain the effective antibody for HA and NS of SIV-H1N1/2009 and the commercialized antibodies did not function perhaps because of the sequence specificity of SIV-H1N1/2009. Furthermore, a miRNA may target multiple genes. Therefore, ssc-miR-204 and ssc-miR-4331 might be involved in SIV-H1N1/2009 infection via regulating some host genes. In addition, in order to further verify that ssc-miR-204 and ssc-miR-4331 inhibiting the replication of SIV-H1N1/2009 is due to the interaction with HA and NS respectively, future work should focus on generating the HA or NS mutated SIV-H1N1/2009 viruses, in which the binding sites for ssc-miR-204 or ssc-miR-4331 are mutated.

## 4. Materials and Methods

### 4.1. Cells Culture

New-born pig trachea (NPTr) cells were established following serial culture of primary cells derived from trachea tissues [48]. NPTr cells were cultured and maintained in Dulbecco’s modified Eagle’s medium (DMEM; HyClone, Logan, UT, USA) supplemented with 10% heat-inactivated fetal bovine serum (FBS; Trans Serum ^TM^, Beijing, China) at 37 °C in a humidified 5% CO_2_ incubator (Sanyo, Osaka, Japan). All cells were confirmed to be free of mycoplasma for the entire study.

### 4.2. Virus Infection

The SIV-H1N1/2009, A/swine/Nanchang/F9/2010(H1N1) (GenBank accession no. JF275925-JF275932), was isolated in swine herds in 2011 by our lab [17]. The H5N1 strain (A/duck/Hubei/Hangmei01/2006(H5N1), GenBank accession no. EU594346-EU594353) and H9N2 strain (A/duck/Hubei/W1/2004(H9N2), GenBank accession no. DQ465397-DQ465404) were also isolated by our lab [49,50]. All viruses were propagated in 9-day-old embryonated chicken eggs and stored at −80 °C. 

NPTr cells planted in 12-well plates were washed with phosphate-buffered saline (PBS) and then infected with SIV-H1N1/2009 or H9N2 influenza A virus at a multiplicity of infection (MOI) of 0.01. After one hour, the cells were re-suspended in DMEM with 0.5 μg/mL of L-tosylamide-2-phenylethyl chloromethyl ketone (TPCK)-treated trypsin (Sigma, St. Louis, MO, USA), and collected at the indicated time points post-infection. All experiments with H5N1 influenza A virus were performed in Animal Biosafety Level 3 laboratory. NPTr cells were infected with H5N1 influenza A virus at a MOI of 0.001, and maintained in DMEM free of TPCK-treated trypsin.

### 4.3. Plasmid Construction

The genomic RNA of SIV-H1N1/2009 was extracted and then reverse transcribed by influenza virus specific-U12 to synthesize single strand cDNA. The viral PB1, PB2, PA, HA, NA, NP and NS genes harboring the target sites of potential miRNAs were PCR-amplified from the cDNA and inserted into the luciferase reporter vector pmirGLO (Promega, Madison, WI, USA). The viral HA and NS mutant constructs were generated by introducing four point mutations at target regions into the corresponding wild-type constructs. All plasmids were verified by DNA sequencing and all primers were listed in Table 3.

### 4.4. Ssc-miR-204 and ssc-miR-4331 Mimics and Inhibitor

MiRNA mimics (double-stranded RNA oligonucleotides), inhibitors (single-stranded RNA oligonucleotides), and their scrambled oligonucleotide negative controls (NC) were commercially synthesized by GenePharma (Shanghai, China). The sequences of them were listed in Table 4.

### 4.5. Transfection 

All of the miRNA mimics or inhibitors were transfected into NPTr cells at 30 nM plus with 100 ng corresponding reporter plasmids per well for dual-luciferase reporter assays or at 60 nM/well individually for other assays in 12-well plates. Transient transfections were performed with lipofectamin2000 (Invitrogen, Waltham, MA, USA) according to the manufacturer’s protocol.

### 4.6. Potential miRNAs Prediction and Dual-Luciferase Reporter Assays

Potential miRNAs that targeted the viral genomic RNA of SIV-H1N1/2009 were predicted by RegRNA 2.0 (Available online: http://regrna2.mbc.nctu.edu.tw/detection.html) which was an online server integrating miRanda and miRBase. The criteria for the selection was set as “score ≥ 150.00 and free-energy ≤ −20.00”, as miRanda based on sequence complementarity, free energy calculations of duplex formation, and evolutionary arguments in developing a scoring scheme for identifying the miRNA–mRNA interaction.

To determine whether the predictions of miRNAs–RNA pairs were positive, luciferase assays were performed by using the Dual-Luciferase Reporter Assay System Kit (Promega) following the manufacturer’s protocol. NPTr cells were cotransfected with miRNA mimics or inhibitors and the corresponding pmirGLO reporter plasmids and incubated for 24 h. After that, cells were washed with PBS softly and lysed in Passive Lysis Buffer. The firefly and renilla luciferase activities were measured using the Dual-Luciferase Reporter Assay System. All results were expressed as relative luciferase activity (firefly luciferase/renilla luciferase). Each treatment was performed in triplicate in three independent experiments.

### 4.7. RNA Extraction, Reverse Transcription and qRT-PCR

Total RNA was extracted from the NPTr cells using TRIzol (Invitrogen, Carlsbad, CA, USA) and then digested using DNase I (Ambion, Austin, TX, USA) according to the manufacturer’s instructions. After that, both RNA purity and concentration were evaluated by a NanoDrop spectrophotometer (NanoDrop Technologies, Wilmington, DE, USA). Equal amounts of RNA were reverse transcribed (RT) to cDNA using AMV reverse transcriptase (Takara, Kyoto, Japan) in accordance to the manufacturer’s protocol. The RT primer for genes were oligo(dT) 18 primer and the specific stem-loop RT primers for miRNAs are listed in Table 5. The generated cDNA was used as the template in the following qRT-PCR and the remaining RNA was stored at −80 °C until further use.

The qRT-PCR was performed using FastStart Universal SYBR Green Master (Roche, Mannheim, Germany) on an ABI ViiA7 instrument (Applied Biosystems, Foster City, CA, USA). The 20 μL reactions mixture included 1 μL of cDNA products, 0.5 μL forward primers and reverse primers, 10 μL SYBR green mix, and 8 μL DNase/RNase-free deionized water. All primers were designed using Primer Premier 5.0 software based on the sequences of the corresponding miRNAs in miRBase and mRNAs in GeneBank (Table 5). The relative expression levels of genes or miRNAs were measured in terms of threshold cycle value and normalized to that of internal control GAPDH or U6 within each sample using 2^−ΔΔ*C*t^ method [51]. Each of the PCR reactions was performed in triplicate to guarantee the reproducibility of amplification of each sample.

### 4.8. Western Blotting

Total protein was abstracted from NPTr cells which were collected and lysed with Mammalian Protein Extraction Reagent (Cwbio, Beijing, China) after transfection and/or infection. Then, the concentration of total protein was measured using Bradford Protein Assay Kit (Beyotime, Shanghai, China) following the manufacturer’s instructions. Equal protein quantities were separated on 10% sodium dodecyl sulfate-polyacrylamide gel electrophoresis (SDS-PAGE) and transferred to the nitrocellulose membrane (GE Life Science, Piscataway, NJ, USA). The membranes were blocked in 5% bovine serum albumin (BSA) for 1 h and then incubated with specific primary antibodies for 2 h at room temperature. After being washed three times, the membranes were incubated with relative second antibodies for 1 h. Finally, the protein blots were visualized using enhanced chemiluminescence reagent (Advansta, Menlo Park, CA, USA). The primary antibodies against β-actin and Horseradish peroxidase-conjugated anti-mouse/rabbit secondary antibodies were purchased from Abclonal Technology (Wuhan, China). Rabbit polyclonal antibody against viral NP was purchased from GeneTex, Inc. (San Antonio, TX, USA).

### 4.9. Neuraminidase Activity Measured by a MUNANA Assay

The virus titers of SIV-H1N1/2009 were evaluated by the neuraminidase activity, for the influenza virus neuraminidase can cleave the 2′-(4-methylumbelliferyl)-α-d-*N*-acetylneuraminic acid (MUNANA, Sigma) to yield a fluorescent 4-methylumbelliferyl (4-MU) that can be quantified [52]. Briefly, 2 μL of infection supernatants were added into a 96-well black flat-bottom plate and mixed with 98 μL of 20 μM MUNANA solution containing calcium ion and magnesium ion. After 1 h of incubation at 37 °C, the reaction was terminated by adding 100 μL of stop solution (0.1 M glycine and 25% ethanol with pH 10.7). Fluorescence was measured with EnVision Multimode Plate Reader (PerkinElmer, Waltham, MA, USA) at 365-nm excitation and 450-nm emission wavelengths.

### 4.10. Statistical Analysis

All assays were carried out in triplicate and the results shown were representative of at least three independent experiments. Data were shown as mean ± standard deviation. Statistical analysis was performed using Student’s *t*-test with the Graph Pad Prism 5 software (Graph Pad Software Inc., San Diego, CA, USA). *p* ≤ 0.05 was considered as significant and the asterisks in all figures are defined, * *p* ≤ 0.05, ** *p* ≤ 0.01, *** *p* ≤ 0.001.

## 5. Conclusions

In summary, this work screened the genome-wide prediction of swine miRNAs and SIV-H1N1/2009 interactions, and for the first time found that ssc-miR-204 and ssc-miR-4331 negatively regulated the replication of SIV-H1N1/2009 by targeting viral HA and NS respectively, which provided us valuable insight into the involvement of swine miRNAs in the swine influenza viruses infection.

## Figures and Tables

**Table 1:** Potential microRNAs (miRNAs) targeting the SIV-H1N1/2009 genomic RNA predicted by RegRNA 2.0 (score ≥ 150.00 and free-energy ≤ −20.00). ssc-: *Sus scrofa*.

Genomic RNA	Predicted miRNAs	Free-Energy	Score
PB1	ssc-miR-107	−22.77	155.00
	ssc-miR-504	−20.21	158.00
PB2	ssc-miR-27a	−22.26	151.00
	ssc-miR-181d-5p	−21.26	159.00
	ssc-miR-664-3p	−22.06	156.00
	ssc-miR-769-3p	−20.39	164.00
	ssc-miR-361-3p	−27.73	157.00
	ssc-miR-4334-5p	−22.00	153.00
PA	ssc-miR-146a-3p	−20.01	156.00
	ssc-miR-432-5p	−23.28	159.00
HA	ssc-miR-204	−22.23	163.00
	ssc-miR-365-5p	−20.89	152.00
	ssc-miR-361-3p	−25.01	152.00
	ssc-miR-339-5p	−24.67	152.00
	ssc-miR-4334-3p	−24.13	152.00
NA	ssc-miR-136	−26.18	173.00
NP	ssc-miR-136	−24.75	180.00
	ssc-miR-205	−20.05	155.00
	ssc-miR-204	−22.71	164.00
	ssc-miR-769-3p	−22.61	152.00
NS	ssc-miR-1307	−29.54	160.00
	ssc-miR-4331	−28.03	164.00

**Table 2:** Potential miRNAs targeting the genomic RNA of H5N1 influenza A virus predicted by RegRNA 2.0 (score ≥ 150.00 and free-energy ≤ −20.00).

Genomic RNA	Predicted miRNAs	Free-Energy	Score
PB1	ssc-miR-186	−23.53	157.00
	ssc-miR-196a	−20.32	154.00
	ssc-miR-133b	−23.79	160.00
	ssc-miR-196b-5p	−22.05	158.00
	ssc-miR-339-5p	−21.79	153.00
	ssc-miR-4338	−21.74	172.00
	ssc-miR-129b	−20.61	168.00
PB2	ssc-miR-27b	−22.47	159.00
	ssc-miR-664-3p	−23.23	153.00
	ssc-miR-92a	−30.34	164.00
	ssc-miR-92b-3p	−25.65	156.00
	ssc-miR-1307	−21.08	150.00
	ssc-miR-758	−25.20	152.00
	ssc-miR-194-3p	−29.20	167.00
	ssc-miR-652	−20.41	150.00
	ssc-miR-4334-5p	−22.69	153.00
	ssc-miR-1271	−21.95	153.00
	ssc-miR-1296	−22.36	151.00
PA	ssc-miR-219	−22.93	166.00
HA	ssc-miR-151-5p	−25.20	159.00
	ssc-miR-935	−25.96	168.00
NA	ssc-miR-338	−21.40	162.00
	ssc-miR-218	−26.37	165.00
NP	ssc-miR-145	−22.84	160.00
	ssc-miR-205	−20.68	151.00
	ssc-miR-205	−26.16	150.00
	ssc-miR-124a	−23.21	151.00
	ssc-miR-30a-3p	−22.14	157.00
	ssc-miR-487b	−22.78	152.00
	ssc-miR-219	−20.93	158.00
	ssc-miR-1343	−22.47	155.00
NS	ssc-miR-181a	−21.82	154.00
	ssc-miR-365-5p	−24.76	155.00
	ssc-miR-1307	−29.86	164.00
M	ssc-miR-4336	−20.86	153.00
	ssc-miR-129b	−23.31	157.00

**Table 3:** Primers for plasmids construction used in dual-luciferase reporter assays.

Primers	Sequence (5′–3′)
pmirGLO-HA-F	CTAGCTAGCAGCAAAAGCAGGGGAAAAC
pmirGLO-HA-R	CCGCTCGAGTAGTAGAAACAAGGGTGTTTTTTTC
pmirGLO-HA-mut-F	ATTAATGATATTCCGAAAGAAGTCC
pmirGLO-HA-mut-R	GGACTTCTTTCGGAATATCATTAAT
pmirGLO-NS-F	TACGAGCTCGCAAAAGCAGGGTGACAA
pmirGLO-NS-R	TGCTCTAGATAGTAGAAACAAGGGTGTTTTTTAT
pmirGLO-NS-mut-F	GATATCGAAACTCGGACTCTTGTTG
pmirGLO-NS-mut-R	CAACAAGAGTCCGAGTTTCGATATC
pmirGLO-PB1-F	TACGAGCTCAGCAAAAGCAGGCAAACC
pmirGLO-PB1-R	CCGCTCGAGTAGTAGAAACAAGGCATTTTTTCA
pmirGLO-PB2-F	CCGCTCGAGAGCAAAAGCAGGTCAAATATATT
pmirGLO-PB2-R	TGCTCTAGATAGTAGAAACAAGGTCGTTTTAAAC
pmirGLO-PA-F	TACGAGCTCAGCAAAAGCAGGTACTGATCC
pmirGLO-PA -R	CCGCTCGAGTAGTAGAAACAAGGTACTTTTTTGG
pmirGLO-NA-F	TACGAGCTCAGCAAAAGCAGGAGTTTAAAAT
pmirGLO-NA-R	CCGCTCGAGTAGTAGAAACAAGGAGTTTTTTGAAC
pmirGLO-NP-F	CCGCTCGAGAGCAAAAGCAGGGTAGATAATC
pmirGLO-NP-R	TGCTCTAGATAGTAGAAACAAGGGTATTTTTCCT

**Table 4:** The sequences of ssc-miR-204 and ssc-miR-4331 mimics and inhibitors.

miRNAs	Sequence (5′–3′)
ssc-miR-204 mimics Forward	UUCCCUUUGUCAUCCUAUGCCU
ssc-miR-204 mimics Reversed	GCAUAGGAUGACAAAGGGAAUU
ssc-miR-4331 mimics Forward	UGUGGCUGUGGUGUAGGCCAGC
ssc-miR-4331 mimics Reversed	UGGCCUACACCACAGCCACAUU
mimics NC Forward	UUCUCCGAACGUGUCACGUTT
mimics NC Reversed	ACGUGACACGUUCGGACAATT
ssc-miR-204 inhibitor	AGGCAUAGGAUGACAAAGGGAA
ssc-miR-4331 inhibitor	GCUGGCCUACACCACAGCCACA
inhibitor NC	CAGUACUUUUGUGUAGUACAA

**Table 5:** Primers for qRT-PCR.

Primers	Sequence (5′–3′)
HA1-F	GGGTCAAGAAGGGAGAATGA
HA1-R	AATGGGAGGCTGGTGTTTAT
NS1-F	ACTACTAAGGGCTTTCACTG
NS1-R	CATTTCTGCTCTGGAGGT
NP-F	CCACAAGAGGGGTCCAGATT
NP-R	GGAGATTTCGCTGCACTGAG
H5N1-NP-F	CGTTCAGCCCACTTTCTCG
H5N1-NP-R	ATCGGGTTCGTTGCCTTTT
H9N2-NP-F	AACAGCAGCACAACGAGC
H9N2-NP-R	ACAAGCAGGCAAACAGGA
GAPDH-F	ACCACAGTCCATGCCATCAC
GAPDH-R	TCCACCACCCTGTTGCTGTA
ssc-miR-204-RT	GTCGTATCCAGTGCAGGGTCCGAGGTATTCGCACTGGATACGACAGGCATA
ssc-miR-204-F	TGCGGTTCCCTTTGTCATCCT
ssc-miR-204-R	CAGTGCAGGGTCCGAGGT
ssc-miR-4331-RT	GTCGTATCCAGTGCAGGGTCCGAGGTATTCGCACTGGATACGACGCTGGCC
ssc-miR-4331-F	TGCGGTGTGGCTGTGGTGTAG
ssc-miR-4331-R	CAGTGCAGGGTCCGAGGT
U6-RT	CGCTTCACGAATTTGCGTGTCAT
U6-F	GCTTCGGCAGCACATATACTAAAAT
U6-R	CGCTTCACGAATTTGCGTGTCAT
